# Large language models for tympanostomy patient education: readability and guideline adherence

**DOI:** 10.1007/s00405-026-10612-2

**Published:** 2026-09-16

**Authors:** Shreeya Bahethi, Hetal Lad, Shrey Shah, Sudeepti Vedula, Brian Manzi

**Affiliations:** 1 Department of Otolaryngology-Head and Neck Surgery, Hackensack University Medical Center, Hackensack, NJ 07662 USA; 2 Department of Otolaryngology-Head and Neck Surgery, Rutgers New Jersey Medical School, 90 Bergen Street, Suite 8100, Newark, NJ 07103 USA

**Keywords:** Artificial intelligence, Large language models, Tympanostomy, Clinical practice guidelines, Patient education, Readability

## Abstract

**Purpose:**

To evaluate the accuracy and readability of large language model (LLM)-generated patient education materials regarding tympanostomy tube placement.

**Methods:**

Over a two-month period, ChatGPT 4o, Gemini 2.5 Flash, and Google Search AI were prompted daily using long-form and layered prompt formats covering typical concerns regarding tympanostomy. Responses were scored on a 12-point rubric adapted from the AAO-HNS Clinical Practice Guidelines (CPG), assessing diagnostic accuracy, procedural clarity, and postoperative care. Readability was evaluated using Flesch Reading Ease and Flesch-Kincaid Grade Level. For each model and prompt type, average scores and variability were analyzed with 95% confidence intervals. Between-model differences were tested with Welch’s *t*-tests and Cohen’s d; temporal trends were analyzed via linear regression.

**Results:**

For long-form outputs, Google Search AI and Gemini 2.5 Flash demonstrated the highest mean CPG adherence (96.4% and 96.6%, respectively), both significantly exceeding ChatGPT 4o (84.3%; both *P* < .001; Cohen’s *d* ≈ 2.05). For layered prompt sessions, Google Search AI again demonstrated the highest adherence (91.7%), followed by Gemini 2.5 Flash (88.1%) and ChatGPT 4o (76.2%). Guideline adherence was temporally stable across all models (*P* > .05 for most). All outputs exceeded the recommended sixth-grade reading threshold (mean FKGL, 9.4 for long-form; 10.6 for layered), with no statistically significant readability differences between prompt strategies.

**Conclusions:**

Google Search AI demonstrated the highest guideline concordance, though all models produced material too complex for typical patient comprehension. Structured prompting enhances clinical accuracy, but readability remains a key barrier to accessibility.

## Introduction

Tympanostomy tube placement is among the most commonly performed pediatric surgical procedures in the United States, with an estimated 667,000 procedures performed annually [1]. The AAO-HNS Clinical Practice Guideline provides evidence-based recommendations regarding indications, perioperative counseling, and postoperative management [2]. Adherence to these guidelines supports consistency and quality of care.

Large language models (LLMs) are increasingly used by patients and caregivers to obtain medical information. While these systems may improve accessibility to health information, concerns persist regarding their reliability, guideline adherence, and clinical accuracy across platforms [3, 4]. Variability in LLM performance has been documented across multiple medical specialties, yet data specific to pediatric otolaryngology remain limited.

Readability is equally critical to patient-centered care. National recommendations advise that patient education materials be written at or below a sixth-grade reading level to promote health equity [5, 6]. Prior otolaryngologic literature has demonstrated that online patient education materials frequently exceed this threshold [7]. Whether AI-generated tympanostomy counseling aligns with readability standards is not yet established.

Prompt structure has been shown to influence LLM output quality and reasoning performance [8]. Its impact on guideline adherence in pediatric otolaryngology has not been systematically evaluated. This study assesses CPG adherence and readability of AI-generated tympanostomy counseling across three platforms and two prompt strategies.

## Materials and methods

### Study design and setting

This prospective comparative observational study was conducted from August 1 to October 11, 2025. Three publicly available AI platforms were evaluated: ChatGPT 4o, Gemini 2.5 Flash and Google Search AI. Institutional review board approval was not required. This study did not involve human participants, patient data, or animal subjects, as it exclusively evaluated publicly available outputs generated by artificial intelligence platforms in response to standardized prompts. Therefore, it was exempt from institutional ethics review board oversight in accordance with institutional policy.

### Prompt strategy

Each platform was queried using two standardized formats. The *long-form* format consisted of a single comprehensive query requesting detailed tympanostomy counseling encompassing indications, procedural explanation, risks and complications, alternatives, postoperative care, water precautions, and follow-up. The *layered* format employed sequential structured prompts addressing each counseling component discretely across four queries per session. Long-form prompts were issued daily (71–72 responses per model; *n* = 215 total). Layered sessions were conducted weekly (7 sessions per model; *n* = 21 sessions; 84 individual responses).

### CPG adherence scoring

Responses were evaluated using a 12-point rubric adapted from the 2022 AAO-HNS CPG on Tympanostomy Tubes in Children. Eight criteria were scored across three domains: diagnostic assessment (Criterion 1, 0–2 points), perioperative counseling (Criteria 2–5, 0–2, 0–2, 0–2, 0–1 points), and postoperative guidance (Criteria 6–8, 0–1 points each). For layered sessions, responses were aggregated prior to scoring. Percent adherence was calculated as (total score/12) × 100.

### Readability assessment

Readability was assessed using the Flesch Reading Ease (FRE) score (0–100; higher = easier; ≥ 70 corresponds to ≤ 6th-grade level) and the Flesch-Kincaid Grade Level (FKGL). For layered sessions, readability was scored per individual response.

### Statistical analysis

Descriptive statistics including mean, standard deviation (SD), and 95% confidence intervals (CI) were calculated for each model and prompt type. Between-group differences were assessed using Welch’s *t*-tests; effect sizes were quantified with Cohen’s *d*. One-way analysis of variance (ANOVA) was used for between-model readability comparisons. Temporal trends were assessed by linear regression. Statistical significance was defined as *P* < 0.05.

## Results

### Study overview

A total of 215 long-form responses and 21 layered prompt sessions (84 individual responses) were analyzed across the 72-day study period.

### CPG adherence

#### Long-form prompts

**Fig. 1:**
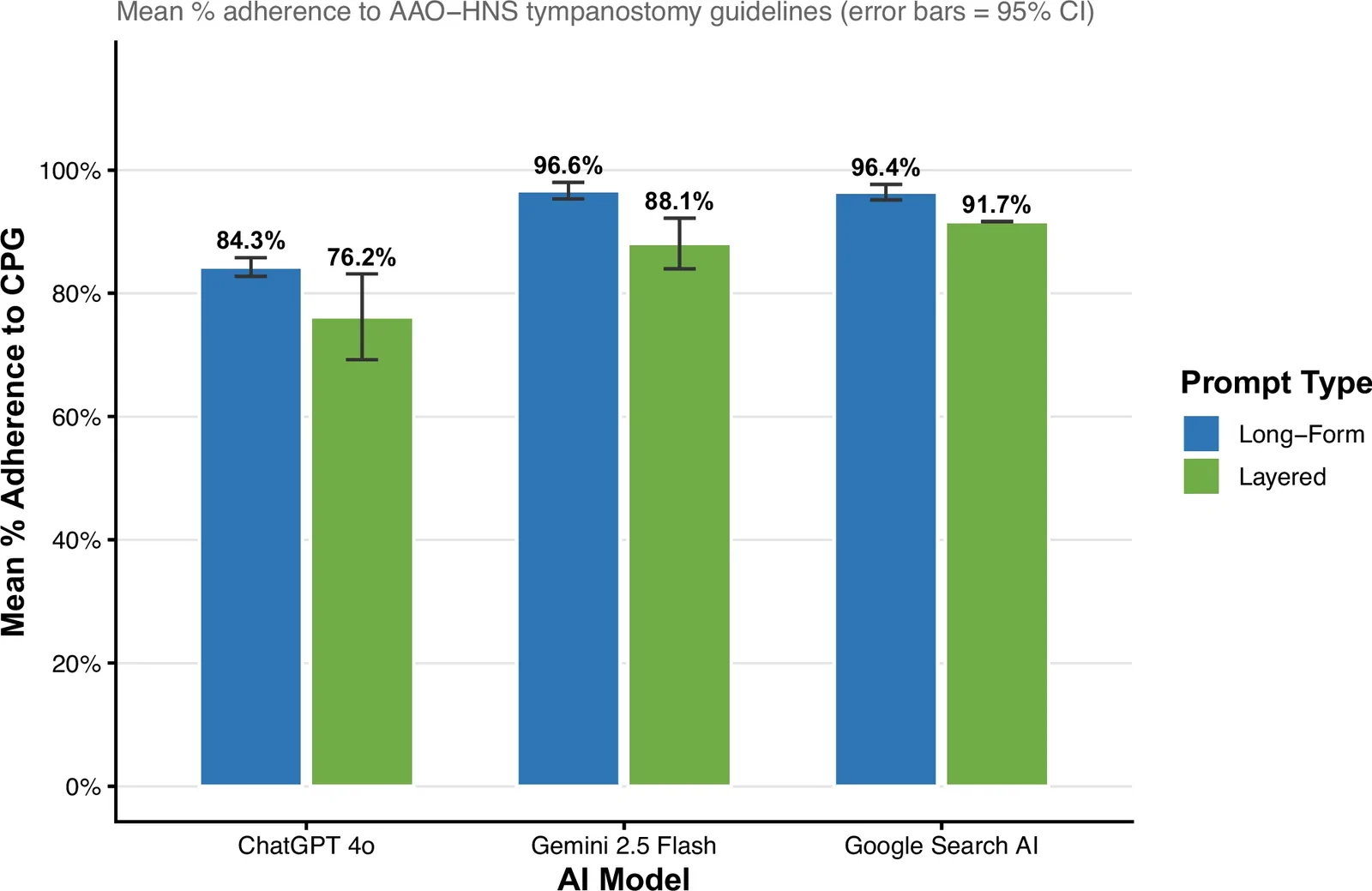
Mean CPG adherence (%) by AI model and prompt type. Error bars represent 95% confidence intervals. Long-form and layered prompt formats shown side by side for each model

Mean CPG adherence across all long-form outputs was 92.5% (95% CI, 91.4–93.6%). Google Search AI and Gemini 2.5 Flash demonstrated equivalent and highest adherence at 96.4% (95% CI, 95.1–97.7%; SD, 5.4) and 96.6% (95% CI, 95.3–98.0%; SD, 5.7), respectively. ChatGPT 4o demonstrated significantly lower adherence at 84.3% (95% CI, 82.8–85.8%; SD, 6.4). Pairwise comparisons confirmed significant differences between ChatGPT 4o and Google Search AI (difference, + 12.1 percentage points; *P* < 0.001; Cohen’s *d* = 2.06) and between ChatGPT 4o and Gemini 2.5 Flash (difference, + 12.4 points; *P* < 0.001; Cohen’s *d* = 2.04). No significant difference was observed between Google Search AI and Gemini 2.5 Flash (*P* = 0.80; Fig. 1, Table 1).

**Table 1:** CPG adherence by AI model and prompt type

Model	Prompt Type	n	Mean % Adherence	SD	95% CI
ChatGPT 4o	Long-Form	71	84.3	6.4	82.8–85.8
Gemini 2.5 Flash	Long-Form	72	96.6	5.7	95.3–98.0
Google Search AI	Long-Form	72	96.4	5.4	95.1–97.7
ChatGPT 4o	Layered	7	76.2	7.5	69.3–83.1
Gemini 2.5 Flash	Layered	7	88.1	4.5	84.0–92.2
Google Search AI	Layered	7	91.7	0.0	91.7–91.7

*CI* confidence interval; *CPG* clinical practice guideline; *SD* standard deviation. Adherence scored on a 12-point rubric adapted from the 2022 AAO-HNS CPG on Tympanostomy Tubes in Children. Percent adherence = (total score/12) × 100

#### Layered prompts

Mean CPG adherence across layered sessions was 85.3% (95% CI, 81.5–89.1%). Google Search AI demonstrated the highest and most consistent adherence at 91.7% (SD, 0.0), followed by Gemini 2.5 Flash at 88.1% (95% CI, 84.0–92.2%; SD, 4.5) and ChatGPT 4o at 76.2% (95% CI, 69.3–83.1%; SD, 7.5). The rank order of model performance was consistent across both prompt formats (Fig. 1, Table 1). Pairwise model differences across both prompt conditions are illustrated in Fig. 4.

### Readability

#### Long-form prompts

**Fig. 2:**
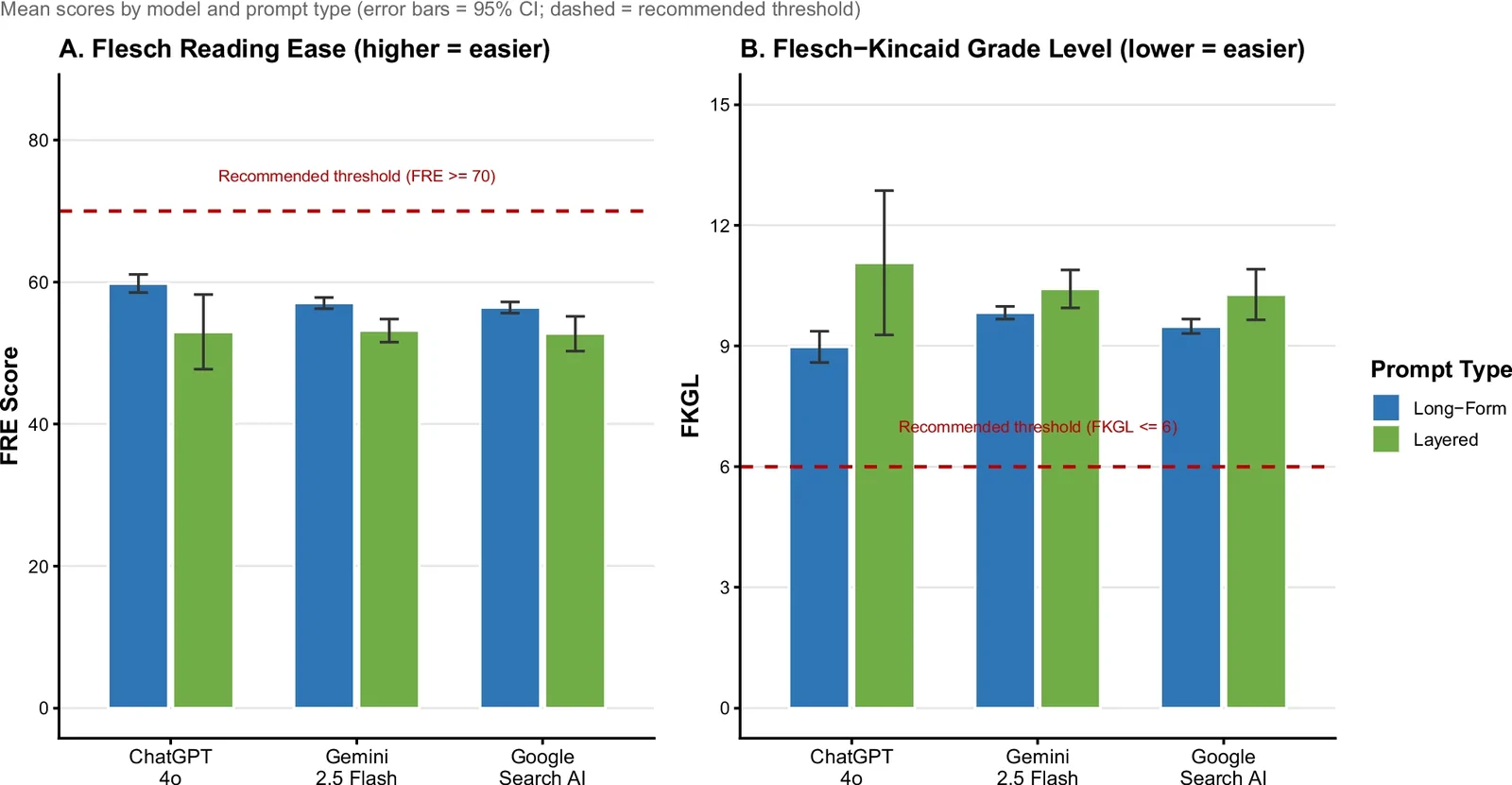
Readability of AI-generated tympanostomy counseling by model and prompt type. Panel **A**: Flesch Reading Ease scores (higher = easier). Panel **B**: Flesch-Kincaid Grade Level scores (lower = easier). Dashed red line indicates recommended patient education threshold (FRE ≥ 70; FKGL ≤ 6). Error bars represent 95% confidence intervals

Mean FRE across long-form outputs was 57.75 (95% CI, 57.16–58.34), corresponding to a “fairly difficult” reading level. Mean FKGL was 9.43 (95% CI, 9.28–9.59), equivalent to a ninth-grade reading level. ChatGPT 4o produced the most readable outputs (FRE, 59.8; FKGL, 9.0), followed by Google Search AI (FRE, 56.4; FKGL, 9.5) and Gemini 2.5 Flash (FRE, 57.0; FKGL, 9.8). Between-model differences were statistically significant (FRES: *F* = 8.52, *P* < 0.001; FKGL: *F* = 11.25, *P* < 0.001). All outputs exceeded the recommended sixth-grade threshold (Fig. 2).

#### Layered prompts

**Fig. 3:**
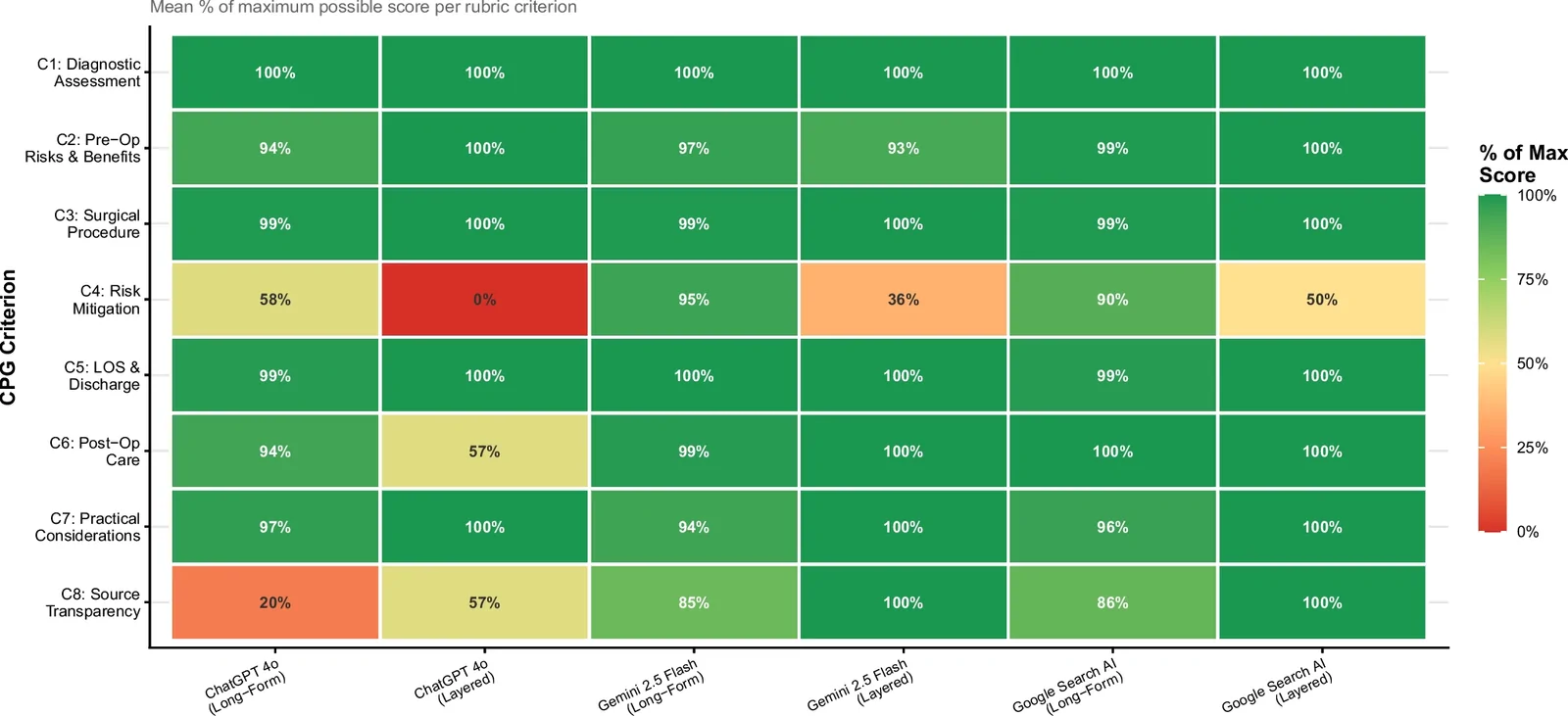
Heatmap of mean CPG criterion adherence by AI model and prompt type. Values represent mean percentage of maximum possible score per rubric criterion. Color scale: red = low adherence, green = high adherence

**Fig. 4:**
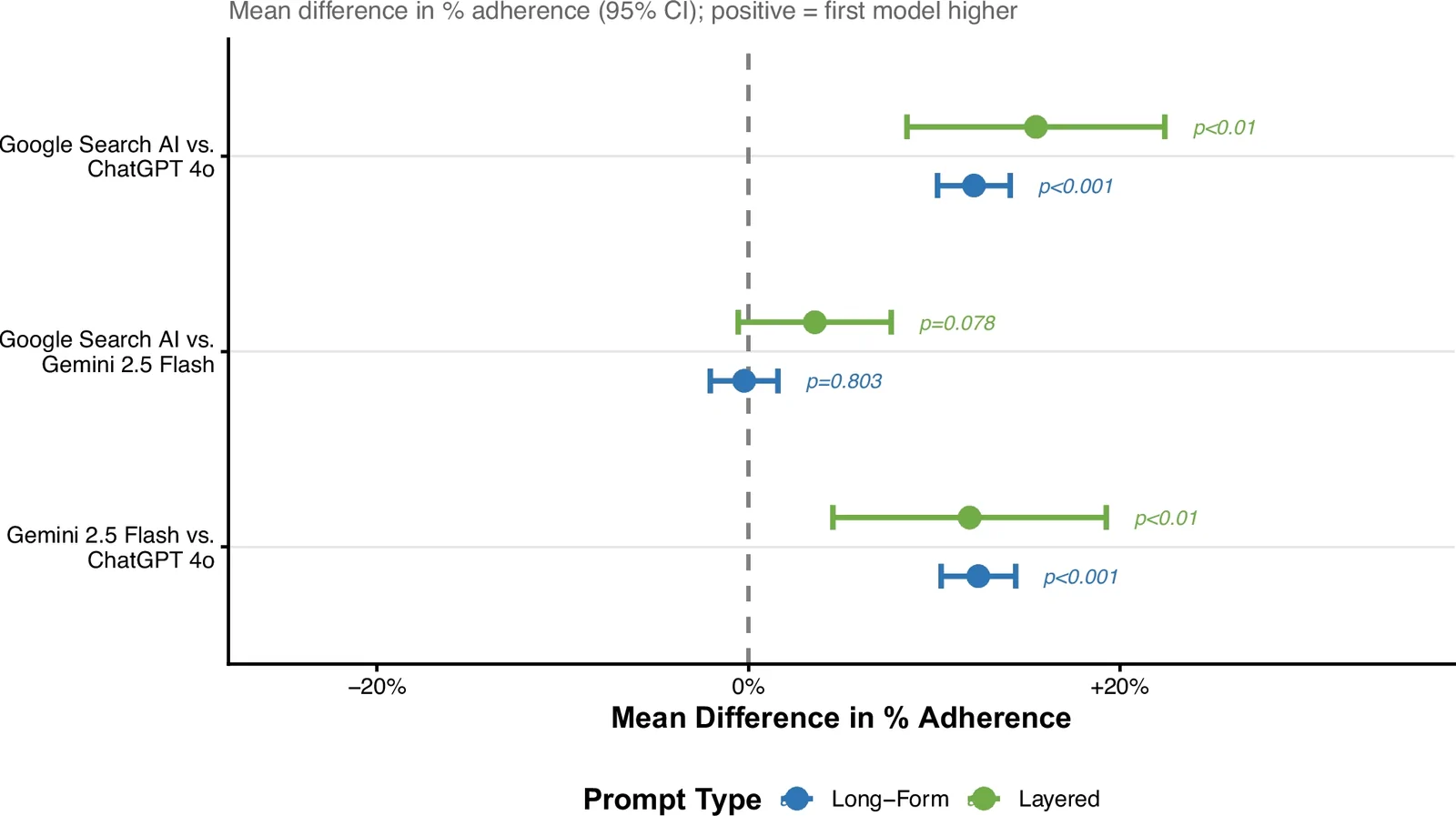
Pairwise comparisons of CPG adherence between AI models by prompt type. Points represent mean differences; horizontal bars represent 95% confidence intervals. Positive values indicate the first-named model scored higher. *P*-values derived from Welch’s *t*-tests

Layered prompt responses produced lower readability than long-form outputs (mean FRE, 52.96 [95% CI, 51.33–54.59]; mean FKGL, 10.59 [95% CI, 10.04–11.14]). ChatGPT 4o demonstrated the greatest variability (FKGL 95% CI, 9.3–12.9), whereas Gemini 2.5 Flash and Google Search AI were more consistent. No model achieved the recommended sixth-grade reading level in either prompt condition (Figs. 2, 3, 4, Table 2).

**Table 2:** Readability scores by AI model and prompt type

Model	Prompt Type	n	Mean FRE (95% CI)	Mean FKGL (95% CI)	Grade Level
ChatGPT 4o	Long-Form	71	59.8 (58.5–61.1)	9.0 (8.6–9.4)	9th
Gemini 2.5 Flash	Long-Form	72	57.0 (56.2–57.8)	9.8 (9.7–10.0)	10th
Google Search AI	Long-Form	72	56.4 (55.6–57.2)	9.5 (9.3–9.7)	9th–10th
ChatGPT 4o	Layered	28	53.0 (47.7–58.3)	11.1 (9.3–12.9)	11th
Gemini 2.5 Flash	Layered	28	53.1 (51.5–54.8)	10.4 (10.0–10.9)	10th
Google Search AI	Layered	28	52.7 (50.3–55.2)	10.3 (9.7–10.9)	10th

*CI* confidence interval; *FKGL* Flesch-Kincaid Grade Level; *FRE* Flesch Reading Ease. Recommended patient education threshold: FRE ≥ 70 (≤ 6th-grade level). Layered n reflects individual prompt responses; long-form n reflects individual session outputs

### Temporal trends

**Fig. 5:**
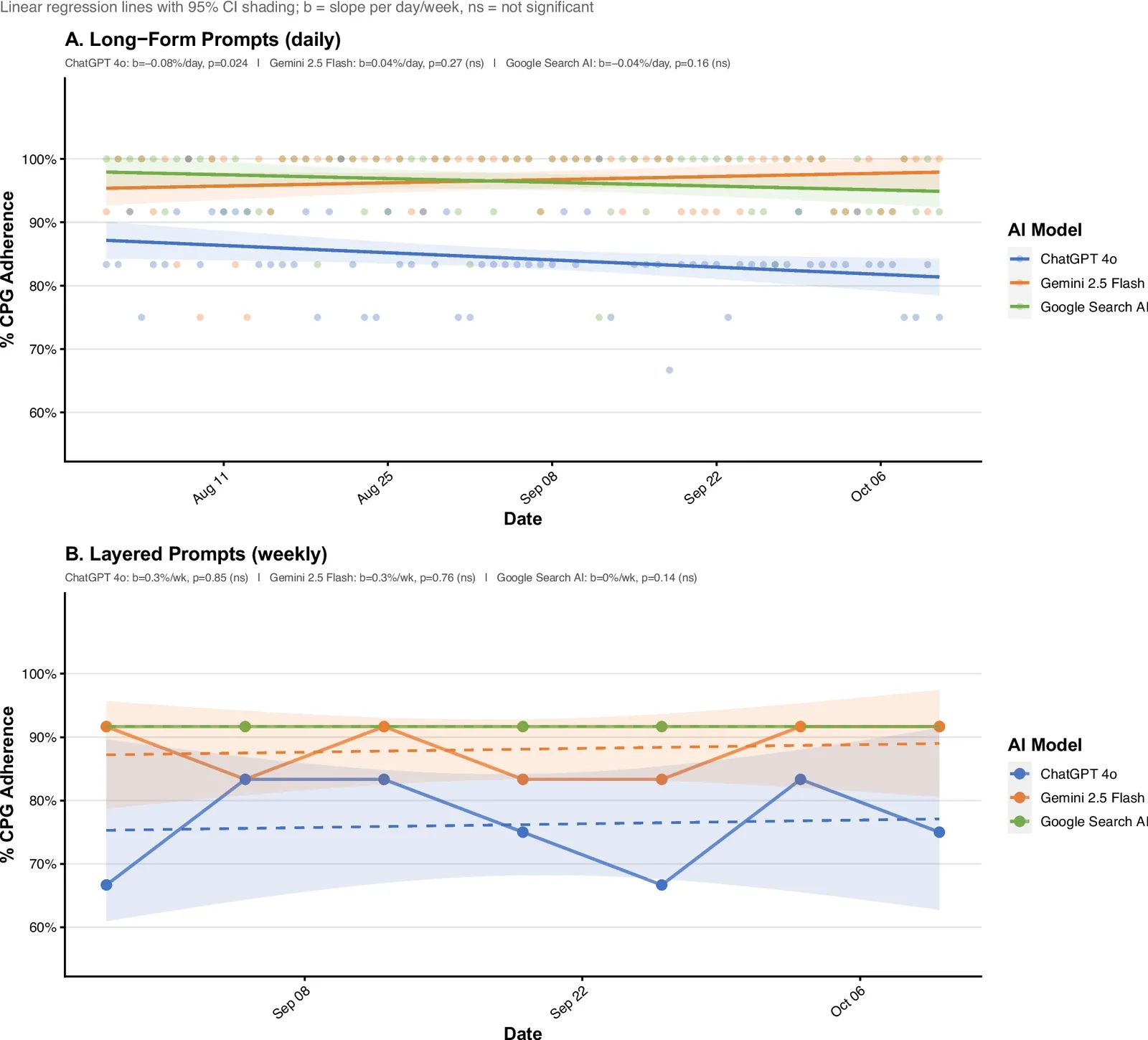
Temporal trends in CPG adherence over the study period. Panel **A**: Long-form prompts (daily data, August 1–October 11, 2025). Panel **B**: Layered prompts (weekly data). Lines represent linear regression fits with 95% confidence interval shading. Regression slope (β) and significance reported in panel subtitles

Linear regression demonstrated no statistically significant change in CPG adherence over the study period for Google Search AI (*β* = − 0.04%/day; *P* = 0.16) or Gemini 2.5 Flash (*β* = + 0.04%/day; *P* = 0.27), indicating temporal stability. A statistically significant but modest negative trend was observed for ChatGPT 4o (*β* = − 0.08%/day; *P* = 0.024), representing an estimated decline of approximately 5.8 percentage points over the 72-day window (Fig. 5).

## Discussion

This study confirms that current LLMs can generate tympanostomy counseling with substantial guideline overlap, providing a useful performance baseline. However, the more pressing clinical question is no longer whether LLMs can produce plausible patient education content, as growing evidence suggests, but rather when they fail, which patient’s face greatest latent risks and how these tools can be safely utilized [3, 4, 9]. Addressing this discrepancy requires a shift from measuring content coverage to evaluating reliability, safety, and equity conscious rehabilitation.

The 12-point adherence gap between ChatGPT 4o and the other two models confirms that AI platforms are not clinically interchangeable, consistent with prior cross-specialty benchmarking [3, 4]. Critically, the CPG rubric measured content presence, not content accuracy. A model providing complete but subtly incorrect risk estimates or fabricated statistics would score well yet pose a clinical hazard. Medical hallucinations, where LLMs generate misleading or fabricated clinical information, have been shown to compromise patient safety and reduce clinician trust, making rubric adherence a necessary but insufficient benchmark for utilization [10, 11].

A critical question this study cannot fully answer, but which future work must address is: under what circumstances, and for which patients, is LLM-generated counseling most likely to cause harm? Several potential risks emerge from this data. ChatGPT 4o’s high output variability individual caregivers may receive responses more complex or less complete than the average. The modest decline in ChatGPT 4o adherence over time suggests performance can shift as architectures are updated without clinical notice [12]. Most consequentially, caregivers with low health literacy, limited English proficiency, or low digital fluency are least equipped to recognize AI errors, seek clarification, or access a supplementary clinical source [5, 6, 13, 14]. In tympanostomy counseling, failure to convey procedure aspects including water precautions, tube lifespan, or follow-up expectations carries direct postoperative consequences.

The implications for health equity are significant, and demographic bias represents further concern. Recent evidence demonstrates that LLMs produce systematically different clinical recommendations based on race, socioeconomic status, and housing status [15]. If similar patterns extend to surgical counseling, families from minority or low-income backgrounds may receive qualitatively inferior postoperative guidance due to training data biases rather than clinical differences. Given that tympanostomy disproportionately affects children from lower-income households with higher otitis media burden, this population is simultaneously the most frequent recipient of tympanostomy counseling, and among the most vulnerable to AI-related disparities. The premise that AI democratizes health information access requires validation across demographic groups before it can be accepted [16, 17].

All models produced outputs exceeding recommended literacy thresholds regardless of prompt strategy, averaging ninth- to tenth-grade reading levels against a sixth-grade standard [5, 6]. The independence of adherence and readability outcomes confirms these are distinct design targets. Low health literacy is independently associated with poorer surgical risk comprehension and worse postoperative outcomes, and our findings suggest AI-generated counseling perpetuates the same literacy gap documented in traditional otolaryngologic patient education materials [7, 13]. Readability improvement likely requires explicit grade-level constraints embedded in prompt design rather than better guideline coverage.

The finding that layered prompting required multi-step iterative queries introduces an equity concern: the quality of AI-generated information becomes a function of digital literacy rather than clinical need [18, 19]. Embedding standardized prompt templates within clinical portals or patient-facing interfaces would remove this burden from caregivers and reduce differential information access.

Taken together, these findings support a human-in-the-loop integration model in which LLM-generated content functions as a clinician-reviewed draft rather than an autonomous patient resource. Responsible utilization should incorporate automatic readability scoring, grade-level constraints, and structured testing to identify failure conditions before clinical use [20].

The temporal stability of Google Search AI and Gemini 2.5 Flash adherence over 72 days is reassuring. The modest decline in ChatGPT 4o emphasizes that LLM architectures update continuously without clinical announcement, necessitating ongoing prospective performance monitoring analogous to post-market medical device surveillance [12, 20].

Limitations include a rubric-based scoring approach that detects content presence but not factual accuracy or hallucination. The layered dataset comprised seven weekly sessions per model, limiting pairwise statistical power. Readability metrics do not capture individual comprehension, cultural context, or multilingual accessibility, and findings may not generalize beyond the three platforms evaluated during this study window.

## Conclusion

Google Search AI demonstrated the highest adherence to AAO-HNS tympanostomy clinical practice guidelines across both long-form and layered prompt formats, with Gemini 2.5 Flash performing comparably. ChatGPT 4o demonstrated significantly lower adherence. All models generated content exceeding recommended patient literacy levels regardless of prompt strategy. Structured prompting enhances guideline completeness but does not improve readability. LLMs show promise as clinician-supervised drafting tools; however, integration of explicit readability guidelines will be essential before these platforms can be utilized for equitable patient-facing use. 
